# What’s on the menu?: metabolic constraints in the pancreatic tumor microenvironment

**DOI:** 10.1172/JCI191940

**Published:** 2025-07-15

**Authors:** Colin Sheehan, Alexander Muir

**Affiliations:** Ben May Department for Cancer Research, University of Chicago, Chicago, Illinois, USA.

## Abstract

The tumor microenvironment (TME) of pancreatic ductal adenocarcinoma (PDAC) is composed of a dense stromal compartment and is poorly vascularized, resulting in limited nutrient delivery. As a result, PDAC cells must adapt to cope with the metabolic stresses brought on by TME nutrient limitation. In this article, we first review recent studies that have provided quantitative measurements of nutrient levels in the PDAC TME. These studies have provided a new understanding of the nutrient limitations and metabolic stresses that occur in PDAC. We next discuss the adaptive strategies employed by PDAC in response to TME nutrient limitation. We propose that PDAC adaptations to metabolic stress can be generalized into four categories: (a) cutting down on metabolic costs by recycling metabolites and suppressing nonessential processes, (b) upregulating biosynthetic pathways to meet TME metabolic demands, (c) supporting essential metabolic processes with alternative fuel sources, and (d) dampening antiproliferative and cell death responses that nutrient limitation normally triggers. Improving our understanding of the nutrient limitations within the TME, and the adaptations cells employ to cope with these stresses, provides a more complete picture of PDAC biology and reveals new opportunities for therapeutic targeting of this disease.

## Introduction

Pancreatic ductal adenocarcinoma (PDAC) is among the deadliest cancer diagnoses, with an average 5-year survival rate of 13% (1). This poor prognosis results from a lack of effective treatment options, as the vast majority of patients with PDAC are unresponsive to current modes of chemotherapy, immunotherapy, and radiation treatment (2, 3). Multiple aspects of PDAC biology likely contribute to therapy resistance, although the tumor microenvironment (TME) is thought to play a central role (4, 5). The TME within PDAC is unique among human cancers, with two notable histological features. First, PDAC tumors possess a remarkably high stromal composition, comprising up to 90% of the tumor area (6). Second, across human cancers, PDAC possesses the lowest amounts of supportive vasculature (7), and intercellular signaling within the PDAC TME has been found to actively suppress the formation of new blood vessels (8, 9). Even among the vessels that are present, only a minority (~30%) appear to be functionally active with measurable perfusate (9). Together, increased stroma and decreased vasculature profoundly reduces perfusion throughout PDAC tumors, to the extent that limited perfusion has been explored as a diagnostic marker of the disease (10, 11). Disrupted perfusion is also believed to contribute to therapy resistance by reducing drug delivery to the tumor (9, 12), promoting a drug-resistant cell state (13, 14), and enabling immune evasion (15–18).

While limited perfusion reduces the efficacy of current PDAC therapies, it also presents unique challenges for the cancer cells that reside there. Disruptions in perfusion not only impact the tumoral access to drugs, but also nutrients. This, coupled with the substantial metabolic activities of resident tumor cells, results in nutrient levels in the TME being distinct from those of healthy tissues. Abnormal nutrient availability in the TME constrains cellular metabolism, and PDAC cells must develop adaptations to survive and grow under these conditions. A complete understanding of both the metabolic constraints and cellular responses to the TME has the potential to reveal new drug targets for managing this disease, leveraging limitations intrinsic to the TME to generate therapeutic index (19–21). To determine how PDAC cells adapt to metabolic stress in the TME, we must first describe exactly what types of nutrient limitations these cells encounter. In the first section of this Review, we discuss metabolic constraints that have been identified thus far in the PDAC TME.

## Nutrient availability in the PDAC TME

**Figure 1:**
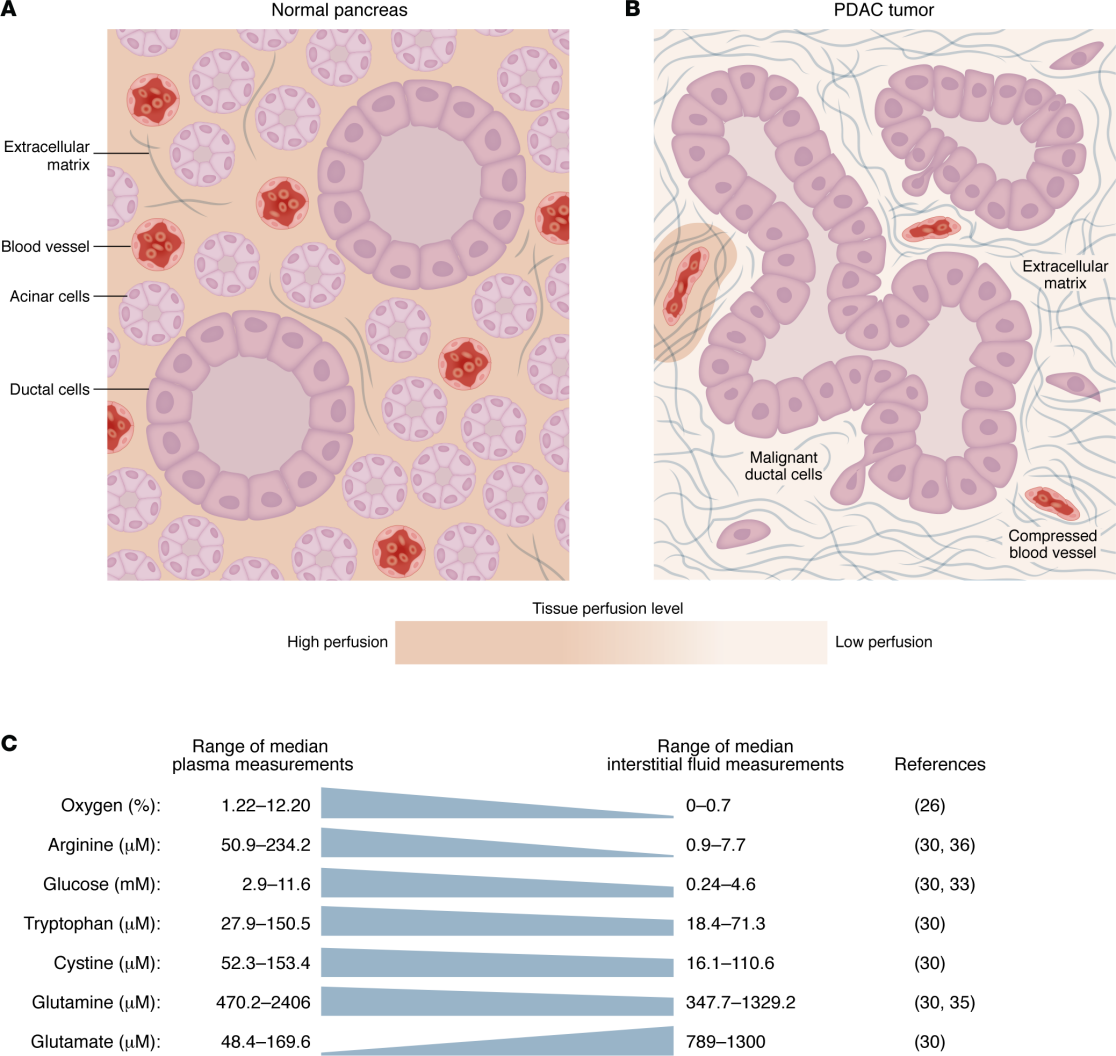
Snapshot of the PDAC nutrient microenvironment. Illustrated cross-sections of (**A**) normal pancreas and (**B**) PDAC with their major structural features highlighted. Differences in the level of perfusion are indicated by the background color gradient. (**C**) Plasma and murine PDAC interstitial fluid concentrations of significantly depleted and accumulated metabolites in the PDAC TME.

Compromised perfusion is expected to have profound consequences for the nutrient supply in PDAC. Recently, advances in sampling tumor interstitial fluid (TIF) (22–24), the local perfusate of the TME that carries metabolites to tumor-resident cells, and mass spectrometry-based metabolomics approaches (25) have provided substantial improvements upon our understanding of metabolite levels in vivo. Below, and in Figure 1, we discuss metabolites that have been identified through these efforts as limiting metabolism in the PDAC TME. It should be noted that most of these measurements were performed utilizing tissue centrifugation-based isolation of TIF. As TIF isolation by centrifugation does not require specialized equipment, this method has become commonly used to measure TME nutrient availability (22). However, TIF isolation by centrifugation must be carefully performed to ensure that prolonged tissue ischemia and/or tumor lysis do not lead to contamination of interstitial fluid with intracellular material (26). Therefore, some caution is warranted in interpreting the results from these studies. Other techniques exist to measure local nutrient availability in tumors (27, 28), and further studies using these complementary approaches will be valuable for determining nutrient availability in the TME.

### Oxygen.

Oxygen is a fundamental component of cellular biochemistry, serving as a terminal electron acceptor for enzymes involved in diverse cellular processes, including oxidative phosphorylation, nucleotide biosynthesis, and protein folding (29, 30). Oxygenation is also one of the most well-studied chemical features of solid tumors. Over two decades ago, oxygen tensions within patient PDAC tumors were first measured using polarographic microelectrodes. These fundamental studies indicate that the median oxygen pO_2_ values of PDAC tumor samples can range from 0 to 5.3 mmHg (0%–0.69%) (31), well below the oxygen tension of healthy tissues, placing PDAC among the most hypoxic of human tumors (32). This low level of oxygen within PDAC tumors can limit the rates for several oxygen-using enzymes (29, 30). Thus, hypoxia is a major constraint on cellular metabolism in the PDAC TME.

### Glucose.

Glucose is a critical nutrient for many metabolic processes, including serving as a carbon source for anabolic growth, generating reducing potential for enzymatic reactions, and supporting protein glycosylation (33). One of the early applications of mass spectrometry–based metabolomics in PDAC analyzed the levels of metabolites in whole PDAC tumors and benign adjacent pancreas samples from the same patient. This analysis revealed that glucose levels are reduced approximately 50% in PDAC relative to the adjacent pancreas (34). Although whole tumor extracts cannot be used to determine extracellular or TME concentrations of a metabolite, these analyses of overall abundance suggest the possibility that levels of a metabolite, such as glucose, are limiting the in the TME. Subsequently, additional studies analyzing glucose content in interstitial fluid from murine models of PDAC also indicate some degree of glucose depletion. The extent of depletion appears to depend on the particular PDAC model under investigation. In genetically engineered mouse models (GEMMs) of PDAC, interstitial fluid analyses indicate a modest 50% depletion of glucose relative to plasma (35). This level of depletion is not below the cellular affinity for glucose, and thus glucose depletion is likely not a metabolic constraint in these models (36, 37). However, in orthotopic allograft models, interstitial fluid levels of glucose can be depleted nearly 8-fold from the plasma to approximately 0.6 mM (38). At this concentration, glucose has been shown to limit cellular uptake and growth (36, 37). Thus, glucose deprivation is likely a common metabolic feature of the PDAC TME though the extent is context dependent.

### Glutamine.

Glutamine is another important nutrient, serving as both a carbon and nitrogen donor to fuel anabolic metabolism of amino acids, nucleotides and redox cofactors like glutathione (39). Analyses of whole tumor extracts in both human and murine PDAC specimens have found that the amino acid glutamine is significantly decreased in PDAC relative to benign pancreas (34, 40). However, analysis of interstitial fluid samples suggest that glutamine levels in the TME are only modestly depleted in certain model systems. In GEMM PDAC models, glutamine levels in the interstitial fluid are approximately 0.8 mM, which is not depleted relative to levels in the plasma (35). In contrast, in orthotopic transplant models of PDAC, glutamine is depleted to approximately 50% of the plasma concentration (41). Thus, there is a discrepancy between whole tumor analysis, which suggests glutamine is highly depleted in PDAC, and interstitial fluid analyses, which suggest glutamine is only modestly depleted. Further work will be required to understand if glutamine depletion is a metabolic stress in the PDAC TME and for which models and patients this stress is relevant.

### Arginine.

Arginine is a nonessential amino acid with important roles in protein synthesis, urea cycle metabolism, and polyamine biosynthesis (42). Interstitial fluid from GEMMs and orthotopic allograft models of PDAC found that arginine is the most depleted metabolite in the TME, being reduced approximately 60-fold from plasma concentrations to a median concentration of 2.5 μM (35, 43, 44). This concentration is well below the cellular affinity for arginine (45–47) and, thus, may represent a critical metabolic limitation in the TME. Additionally, arginine is unique in that its availability appears specifically tied to the metabolic activity of stromal cells within the TME. Myeloid cells in PDAC tumors upregulate the enzyme arginase-1, which catabolizes extracellular arginine, resulting in its depletion from the TME (18, 43). Direct measurements of TME arginine have only been made in animal models of PDAC, and further work is required to determine if arginine is also depleted in human PDAC tumors. However, several pieces of evidence would suggest that arginine may be limiting in human PDAC. First, arginase-1–expressing myeloid cells are also highly enriched in human PDAC specimens (18, 43). Second, arginine restriction leaves a mutagenic signature in cancer cells. Cancer cells cultured with low levels of arginine preferentially acquire mutations in arginine codons, presumably as a strategy to conserve limited arginine availability (48). Arginine codons are preferentially mutated across many human cancers, including PDAC (48). Finally, arginine deprivation also causes misincorporation of cysteine in place of arginine during protein translation (49). Arginine-to-cysteine mistranslated proteins are commonly found in human PDAC specimens (49). Thus, these data are consistent with arginine restriction in human PDAC, which could limit the metabolic activities of tumor cells.

### Tryptophan.

Tryptophan is an essential amino acid that is involved in protein synthesis and serves as a precursor for certain metabolites such as kynurenine and nicotinamide adenine dinucleotide (50). Interstitial fluid analyses from GEMMs of PDAC found that tryptophan is depleted within the TME, at roughly 36% of the normal plasma concentration (35). Similar to arginine, depletion of tryptophan in PDAC is connected with immune signaling, as inflammatory signals in the TME promote expression of indoleamine 2,3-dioxygenase 1 (IDO1), which degrades tryptophan to produce immunomodulatory metabolites like kynurenine (51). Currently, there are not data on extracellular tryptophan availability in human PDAC specimens. However, indirect evidence suggests that tryptophan may be limiting within human tumors. Tryptophan-starved cells misincorporate phenylalanine for tryptophan during protein translation, and tryptophan-to-phenylalanine mistranslated proteins are found in human PDAC samples (52).

### Cysteine and cystine.

Cysteine, and its oxidized dimer, cystine [referred to hereafter as cyst(e)ine to indicate both forms] are nonessential amino acids with critical roles in protein synthesis and redox metabolism, serving as an important source of sulfur for metabolites like glutathione (53). Analyses of levels of cyst(e)ine from both interstitial fluid (35) and whole tumor extracts (54) have found that cyst(e)ine is decreased approximately 50% in both murine and human PDAC samples. Acute exposure of PDAC cells to these physiological cyst(e)ine levels can result in cell death, suggesting that this level of cyst(e)ine may be a metabolic stress in PDAC (54).

### Vitamins.

Vitamins are a set of essential dietary nutrients that emerging evidence suggests can be limiting for PDAC growth. For instance, vitamin B6 is converted by cells into pyridoxal 5′-phosphate, which serves as a cofactor for enzymes involved in amino acid metabolism and nucleotide biosynthesis (55). Interstitial fluid analyses of murine orthotopic allograft PDAC models have found vitamin B6 levels are decreased in the TME (56). These levels are insufficient to support maximal PDAC growth in culture. Furthermore, tumor growth can be stunted by dietary restriction of vitamin B6 (56). Thus, vitamin B6 may also be a limiting nutrient for PDAC growth. It should be noted that some of these metabolites that are limiting for cancer cell proliferation might also be limiting for proper immune cell function within the TME, leading to immune suppression. For instance, vitamin B6 restriction in the TME also impairs natural killer cell activation leading to PDAC immune evasion (56). We refer readers to recent reviews for further information on how TME nutrient availability affects immune cell metabolism and antitumor immunity (57, 58).

### Accumulated metabolites.

Most discussions of metabolic stresses in PDAC TME have focused on metabolites that are depleted from tumors. However, limited perfusion not only affects the delivery of nutrients for resident tumor cells but also affects the removal of metabolic byproducts. Nutrients that are pathologically elevated could also contribute to the metabolic stress experienced by cancer cells in the TME. For example, accumulation of protons in acidic environments can limit cellular metabolism by inhibiting glycolysis (59–61). While quantitative clinical measurements of extracellular pH (pH_e_) have not been made in PDAC, imaging studies with low pH-sensitive insertional peptides suggest that PDAC tumors are also acidic (62–64). Thus, excess accumulation of protons could be a metabolic stress that challenges PDAC cells. Furthermore, several studies have demonstrated that interstitial fluid from PDAC tumors is enriched in metabolites that would commonly be considered metabolic byproducts, such as acetate, glutamate, and glycine (35, 62, 65), sometimes to millimolar concentrations. Accumulation of these metabolites can also affect cellular uptake and metabolism of other nutrients. For example, high extracellular levels of glutamate can impair cyst(e)ine uptake and metabolism in cancer cells (66). Thus, accumulated metabolites in TME may represent an important metabolic challenge to PDAC cells. However, relative to our understanding of the metabolic stress from depleted metabolites in the TME, we can only speculate on how these accumulated metabolites challenge tumor cells.

## Adaptive mechanisms to overcoming PDAC TME metabolic stress

**Figure 2:**
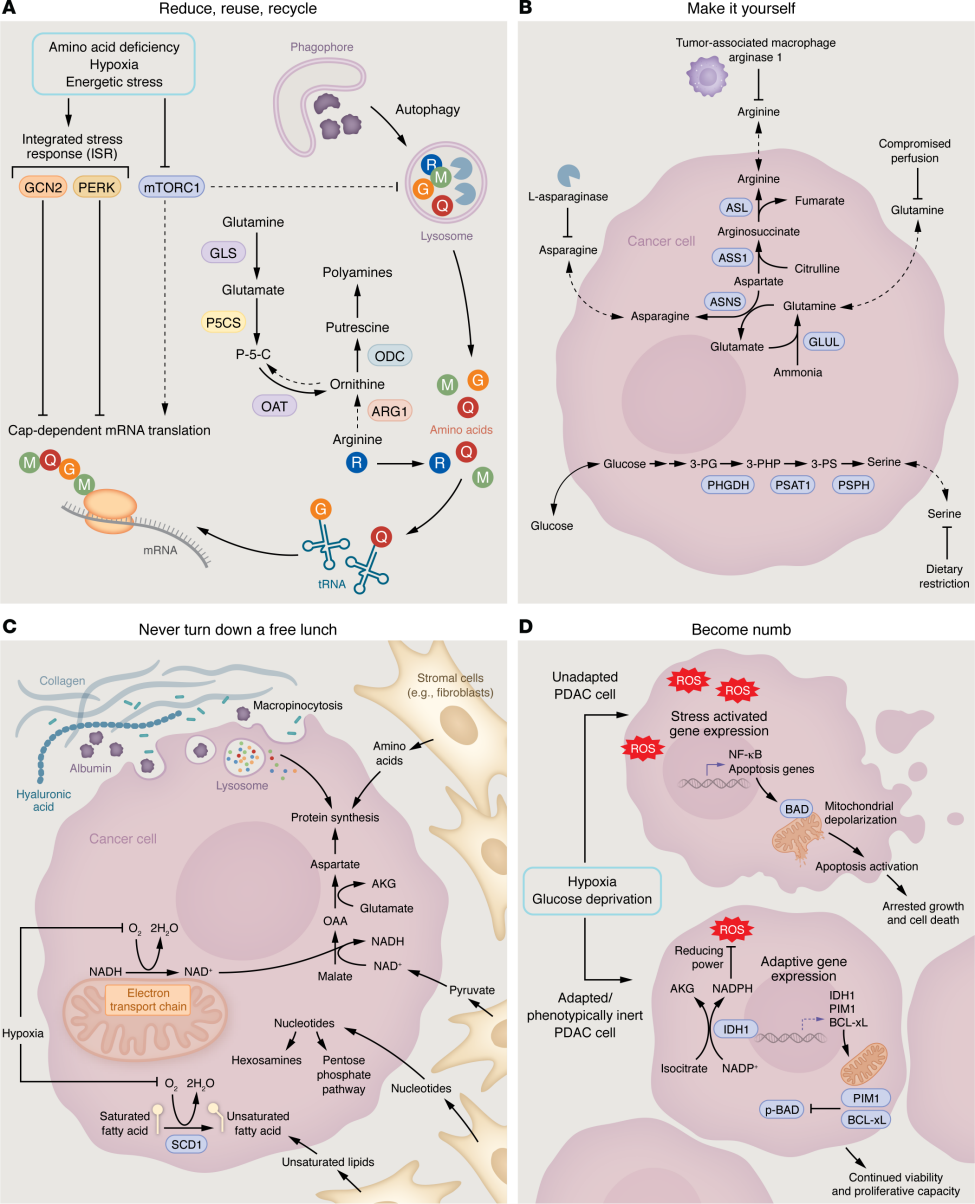
PDAC adaptations to tumor nutrient stress. (**A**) Reduce, reuse, recycle. Nutrient-sensitive regulation allows PDAC to lower cap-dependent mRNA translation to spare amino acids. Reversal of ornithine aminotransferase (OAT) activity allows PDAC to spare limiting arginine. Recycling of macromolecules by autophagy allows PDAC to recapture and reuse metabolites like amino acids. (**B**) Make it yourself. De novo arginine and glutamine synthesis allows PDAC to cope with endogenous starvation of these amino acids in the TME. De novo asparagine and serine synthesis allow PDAC to cope with therapeutic starvation of these amino acids. (**C**) Never turn down a free lunch. Macropinocytosis allows PDAC to acquire amino acids from extracellular protein sources. Metabolic exchange of multiple nutrients with stromal cells can enable PDAC to overcome TME nutrient stress. (**D**) Become numb. Induction of ROS quenching processes, such as isocitrate dehydrogenase 1 (IDH1), allow PDAC to mitigate damage caused by nutrient stress. Dampening of apoptotic signaling allows PDAC to survive nutrient stress that cannot be overcome and would otherwise cause loss of cell viability. AKG, α-ketoglutarate; ARG1, arginase; ASL, arginosuccinate lyase; BAD, BCL-2 associated death promoter; GLS, glutaminase; GLUL, glutamate ammonia ligase; OAA, oxaloacetate; ODC, ornithine decarboxylase; p-BAD, phospho-BAD; P-5-C, pyrroline-5-carboxylate; P5CS, pyrroline-5-carboxylate synthase; PG, phosphoglycerate; PHGDH, phosphoglycerate dehydrogenase; PIM1, proto-oncogene serine/threonine-protein kinase; PS, phosphoserine; PSAT1, phosphoserine aminotransferase; PSPH, phosphoserine phosphatase; SCD1, stearoyl-CoA desaturase; tRNA, transfer RNA.

Pathophysiological nutrient availability in the TME can present a considerable stress for PDAC cells that may limit their ability to grow. Therefore, PDAC cells in tumors must develop adaptations to maximize their fitness in this challenging environment. Many adaptations fit into general themes. Here, we propose four adaptive programs that PDAC cells utilize to cope with nutrient stress in TME: (a) reducing metabolic costs by recycling metabolites and suppressing nonessential processes, (b) upregulating biosynthetic pathways to meet TME metabolic demands, (c) supporting essential metabolic processes with alternative fuel sources, and (d) dampening antiproliferative and cell death responses triggered by nutrient limitation. Below and in Figure 2, we describe how these adaptive mechanisms allow PDAC to cope with TME nutrient stress.

### Reduce, reuse, and recycle.

When faced with limited stores of a metabolite, one of the most important responses by the cell is to reduce consumption of that metabolite. The most widely recognized example of this is the “Pasteur Effect” observed in cells under low-oxygen conditions, in which cells reduce mitochondrial respiration and direct substrates away from the TCA cycle through the action of hypoxia-inducible factors (HIFs) (67, 68). In doing so, oxygen is conserved to support other essential metabolic processes. Protein translation, while required by all cells, is extremely costly, both energetically due to ATP/GTP hydrolysis and due to consumption of amino acids (69, 70). Given that several amino acids are commonly depleted from the TME (Figure 1), adaptations that reduce unnecessary protein translation may be highly advantageous for PDAC. Indeed, PDAC tumors reduce protein synthesis rates approximately 4-fold lower than those of healthy pancreas (71). Furthermore, consistent with a reduction in the energetic demand from translation, the rate of ATP synthesis in PDAC tumors is approximately 5- to 6-fold lower than those of healthy pancreas (71). Given the substantial energetic and amino acids costs of protein translation, PDAC suppression of protein synthesis could be an important adaptation to metabolic stress in the TME.

Given the metabolic costs associated with protein synthesis, cells possess complex regulatory machinery to adjust translation rates based on input from biological sensors of nutrient availability. The majority of mRNA molecules in the cell are translated through an mRNA cap-dependent process (72). However, under stressed conditions, such as when amino acid levels drop, the activities of multiple regulatory kinases are altered to reduce cap-dependent translation. In particular, the mTORC1 complex, whose activity normally promotes mRNA translation, becomes inactivated upon amino acid deprivation (69, 73). Simultaneously, kinases that are part of the integrated stress response (ISR), such as GCN2 and PERK, are activated to suppress translation initiation (74). These pathways are modulated in PDAC tumors, as histological analyses from patient and preclinical cancer models indicate that the ISR is activated while mTORC1 is inactivated in PDAC tumors (13, 75). Furthermore, there is clear evidence that these pathways are functionally important for PDAC progression. Hyperactivation of mTORC1 (13, 76–78), or loss-of-function mutation to GCN2 (79), reduces the ability of PDAC to adapt to amino acid stress. Pharmacological inhibition of mTORC1 can even accelerate tumor growth in murine PDAC models (78). Altogether, these data indicate that limiting nonessential mRNA translation is a critical mechanism by which PDAC tumors adapt to nutrient stress in the TME.

Beyond translation, cells might also rewire metabolic pathways to conserve the availability of limiting metabolites for other essential processes. For instance, as discussed above, arginase-1 activity within the TME results in the depletion of the amino acid arginine, which is essential to support protein synthesis (18, 43). However, beyond protein synthesis, cells also catabolize arginine into ornithine to support the synthesis of polyamines, another essential class of molecules in the cell (80). When arginine is abundant, the enzyme ornithine aminotransferase (OAT) will normally catabolize ornithine to generate glutamate and glutamine. However, in PDAC, where arginine is limiting, this enzyme reverses direction to enable cells to biosynthesize ornithine and polyamines from glutamine (44). This switch enables PDAC to spare arginine by using a more readily available substrate in the TME to produce essential polyamines.

Finally, in addition to reducing consumption of limiting metabolites, PDAC tumors adapt to metabolic stress in the TME by promoting recycling and salvage of limiting nutrients. Lysosomal recycling of cellular macromolecules via autophagy has been found to be constitutively activated and contributes to PDAC progression in animals (81–83). Autophagy is specifically required for PDAC cells to maintain intracellular levels of amino acids and energy homeostasis during acute nutrient starvation (83). In addition to autophagic turnover of macromolecules, PDAC also adapts to TME nutrient stresses by salvaging and reusing limiting metabolites. Glucose and glutamine starvation reduces the ability of PDAC to synthesize hexosamines, which critically support protein glycosylation (84). However, despite the limitation of hexosamine synthesis under metabolic stress, PDAC maintains hexosamine levels by upregulating N-acetylglucosamine kinase, which recycles hexosamines from protein or extracellular matrix turnover, enabling PDAC cell survival under nutrient stress and tumor progression (84, 85). Thus, limiting costly metabolic pathways and recycling crucial nutrients are both adaptations that PDAC uses to spare limiting metabolites in the TME. Exploring how PDAC regulates other metabolically expensive processes in the TME will likely provide new insight into how these tumors adapt to metabolic stress.

### Make it yourself.

In well-perfused tissues, cells meet their metabolic needs through nutrients provided by the circulation. However, in tumors where these nutrients are limiting, cancer cells rely on the ability to make nutrients themselves. For instance, as discussed in the previous sections, arginine is one of the most depleted nutrients from the TME (43). As a nonessential amino acid, arginine can also be biosynthesized de novo from aspartate and citrulline via arginosuccinate synthetase 1 (ASS1) and arginosuccinate lyase activity. PDAC tumors respond to arginine starvation by upregulating ASS1 expression and activating de novo arginine biosynthesis (43). Similarly, glutamine can be either taken up from the microenvironment or synthesized de novo. PDAC cells can also cope with glutamine deprivation by upregulating glutamine biosynthesis through increased expression of glutamate ammonia ligase (GLUL) (86–88). Hence, for many nonessential nutrients, cancer cells may “unlock” the biosynthetic machinery to support their metabolic needs independently.

The ability to synthesize nutrients de novo enables PDAC to cope with both endogenous metabolic limitations as well as those brought on by therapeutic interventions. For instance, therapeutic delivery of the enzyme L-asparaginase results in systemic depletion of the amino acid asparagine; PDAC tumors can readily adapt to this intervention by upregulating de novo asparagine synthesis through asparagine synthetase (ASNS) expression (89–91). There is also great interest in using dietary interventions to cut off nutrients that fuel tumor progression. Similar to asparaginase treatment, efficacy of many of these dietary approaches depends on the ability of tumors to synthesize metabolites de novo (92–95). For example, dietary restriction of the nonessential amino acid serine has been found to limit tumor progression in many animal models of cancer (96, 97), and this dietary intervention is now being evaluated clinically (NCT05078775). However, murine PDAC tumors do not respond to dietary serine restriction (96). PDAC cells expressing oncogenic *KRAS* mutations can overcome this stress by upregulating enzymes involved in de novo serine synthesis and, thus, are not dependent on microenvironmental serine for growth (96). In a second example, caloric restriction (CR) can effectively blunt PDAC growth in preclinical models due to a two-fold effect on lipid metabolism. First, CR limits lipid availability within the TME. Second, CR causes changes in PDAC intracellular signaling such that cells are unable to synthesize lipids de novo. The combination of these effects is necessary for CR to impair PDAC growth, as forced expression of lipid synthetic enzymes blunts the effect of CR in these PDAC models (98). Thus, the ability of PDAC cells to access metabolic programs to synthesize metabolites is a key mechanism by which these tumors evade endogenous and therapeutic nutrient stress.

Individual PDAC cells exhibit both phenotypic heterogeneity and phenotypic plasticity, enabling them to transition between different cell states (99, 100). This heterogeneity and plasticity can be adaptive for PDAC tumors as they adapt to stresses such as chemotherapeutic challenge (101–103). It can also enable PDAC cells to unlock metabolic programs to overcome TME or therapeutic metabolic challenges. For instance, clones of individual PDAC cells isolated from the same PDAC tumor demonstrate dramatically different survival abilities when treated with an inhibitor of cellular respiration such as phenformin (90). Response to respiratory inhibitors depends on the metabolic state of the individual clone prior to treatment. Clones that activate the ISR and the metabolic pathways this stress response pathway controls are more resistant to phenformin than those that do not activate this pathway (90). Interestingly, coculture of phenformin-sensitive clones with phenformin-resistant clones is sufficient to support survival of the sensitive clones during respiratory inhibition (90). Thus, intratumoral heterogeneity leads to diverse metabolic states in PDAC cells that can facilitate adaptation to metabolic challenges, in part by endowing tumor cells with the capability of synthesizing limiting metabolites de novo.

### Never turn down a free lunch.

Metabolic restrictions placed by the TME mean that cancers cells do not always have the luxury of being picky eaters. Instead, they must capitalize on anything that can be potentially used to support their metabolic needs. Thus, a common theme in the metabolic reprogramming of nutrient-stressed cancer cells is switching to alternative fuel sources to support metabolic pathways.

As discussed in the previous sections, glucose can be significantly depleted in the TME. The most well-studied response to glucose limitation in cells is the “Crabtree Effect” (104). The counterweight to the Pasteur Effect, the Crabtree Effect occurs when cells switch from glycolysis to oxidative phosphorylation to support their energetic needs when glucose is limiting. The Crabtree effect potentially explains the observation that oxidative phosphorylation is essential in glucose-limited cancer cultures (13, 105) and a common genetic dependency for tumor growth in vivo (16, 106–111). Beyond the role of glucose in cellular bioenergetics, glucose is involved in several other metabolic pathways, including nucleotide synthesis, hexosamine synthesis, and the pentose phosphate pathway. Crabtree metabolism cannot resolve the constraints placed by glucose deprivation on these metabolic processes. Therefore, PDAC must acquire additional adaptations to cope with glucose deprivation. Recently, glucose-starved PDAC cells and tumors were found to consume uridine, a nucleoside composed of a uracil nucleobase and a ribose sugar (38). This is achieved by upregulated expression of uridine phosphorylase 1 (UPP1), which catabolizes uridine into uracil and ribose-1-phosphate. In the process, the liberated ribose can be further metabolized to support several pathways normally fed by glucose, including the pentose phosphate pathway, nucleotide biosynthesis, and hexosamine biosynthesis. Furthermore, UPP1 appears to be necessary for supporting tumor growth in murine PDAC models (38). Thus, consuming alternative sources of sugars provided by the TME appears to be a critical adaptation by cancer cells for growth in a glucose-depleted nutrient environment.

Cancer cells consume and breakdown macromolecular components of the TME into nutrients to support metabolism. PDAC cells engage in macropinocytosis, a cellular process wherein plasma membrane ruffles are utilized to ingest extracellular material, which is degraded in the lysosome (34, 112). PDAC cells can take up a variety of extracellular materials via this process, including soluble proteins like albumin and extracellular matrix components like collagen (113) and hyaluronic acid (85). Much like the high level of autophagy observed in PDAC, PDAC cells display a high basal rate of macropinocytosis due to oncogenic *KRAS* signaling (114–118). However, macropinocytosis is further upregulated by amino acid starvation and hypoxia and is required for PDAC amino acid homeostasis under these conditions (34, 77, 78, 116, 119, 120). Experiments tracking consumption of labeled macromolecules in PDAC-bearing mice suggest macropinocytosis is active in vivo (121). In animal tumors, the activity of macropinocytosis is also regional. Less perfused tumor regions demonstrate higher degrees of macropinocytosis, consistent with nutrient stress activating this process (119, 120). How macropinocytosis is triggered by nutrient deprivation remains an area of active investigation. However, macropinocytosis activation involves many of the same nutrient-sensitive regulators involved in limiting amino acid use in protein translation, such as mTORC1 (77, 78).

Cancer cells represent a minority of the cellular makeup in tumors. Other cell types within the TME are capable of metabolite exchange and thus can provide support to PDAC cells. One example of this is how cancer cells may cope with hypoxia. Hypoxia causes two known metabolic limitations in PDAC: (a) impaired synthesis of aspartate (122) and (b) inhibition of unsaturated fatty acid synthesis (123). Aspartate is a critical amino acid required both for protein and nucleotide synthesis. Aspartate is largely membrane impermeable (122, 124), and therefore, most cells synthesize this amino acid de novo. Aspartate synthesis requires oxidation through consumption of the cofactor NAD+, which is normally regenerated through cellular respiration. As a result, aspartate synthesis is often limited in hypoxic cells (122, 124–126). PDAC cells can overcome this limitation through metabolite exchange. Stromal fibroblasts can secrete pyruvate, which supports the regeneration of NAD+ in the absence of oxygen (125). PDAC cells thus use pyruvate to enable oxidative synthesis of aspartate even under hypoxic conditions (127). Similarly, the synthesis of unsaturated fatty acids is coupled to oxygen consumption, making PDAC cells dependent on exogenous sources of unsaturated fats to maintain lipid homeostasis (123, 128). Stromal cells again become an important reservoir of lipids that cancer cells may access. Paracrine signals from cancer cells coax lipid-rich pancreatic stellate cells to take on an activated fibroblastic phenotype, resulting in the secretion of lipids for cancer cell consumption in the process (129). Beyond their assisting role during hypoxic stress, stromal cells support many diverse metabolic processes in PDAC cells. Stromal cells provide amino acids (130–135), carbon sources (62), and nucleosides (136–139) to metabolically challenged PDAC cells. They themselves can even be directly consumed and metabolized by PDAC cells (140). Thus, stromal cells provide an important alternative source of nutrients for PDAC cells within the TME.

In addition to acquiring nutrients from alternative sources in the TME, PDAC tumors produce changes in whole body metabolism that can provide nutrients to fuel tumor growth. PDAC tumors influence metabolic activities at distant organ sites, such as the liver in order to trigger metabolite release that supports PDAC cell growth under nutrient limited conditions (141). In another example, weight loss and muscle wasting in patients with PDAC, known as cachexia, has been hypothesized to function as a mechanism of nutrient mobilization to feed tumor growth (142, 143). Consistent with this hypothesis, muscle wasting leads to significant changes in nutrient levels in both the circulation and tumors of PDAC-bearing animals, including an increase in circulatory muscle-derived branched-chain amino acids that is an early marker of PDAC development (144–146). Furthermore, preventing muscle wasting can blunt PDAC progression and extend animal lifespan (146). Thus, nutrient acquisition by cancer cells not only involves the metabolic activities of stromal cells within the TME, but is influenced by the interaction between the tumor and tissues across the whole organism.

The TME possesses many potential nutrients for cancer cells, which PDAC exploits to cope with metabolic constraints. Many of these nutrients are absent from mainstay cell culture models used in PDAC research, which contain only the minimal set of metabolites required for cancer cell growth under ideal conditions. Given the growing evidence that cancer cells capitalize upon diverse sets of underappreciated nutrients in vivo, future studies examining how PDAC metabolizes the full set of TME nutrients will expand our understanding of how PDAC adapts to metabolic stresses in the TME.

### Become numb.

As discussed above, PDAC cells can use several adaptive mechanisms to bypass metabolic limitations in the TME. However, PDAC must also be able to “tolerate” metabolic stresses that cannot be fully overcome. Continued metabolic stress normally activates antiproliferative and cell death pathways that arrest and kill most cell types (147). In contrast, PDAC cells are remarkably tolerant of nutrient stress. For instance, most cancer cells will arrest proliferation and undergo cell death upon anoxic conditions, but PDAC cells are resistant to this stress (148). Similarly, most cancer cells exposed to media lacking key nutrients like glucose and amino acids will die within 36 hours (149). In contrast, PDAC cells can tolerate this stress and remain viable for 48–72 hours (149). Thus, another key adaptation that provides a fitness advantage to PDAC cells in the TME is to dampen or ignore antiproliferative and cell death signals caused by metabolic stress.

Tolerance to nutrient stress can occur by multiple mechanisms. First, PDAC cells can adapt by limiting the amount of cellular damage caused by nutrient limitation. For example, glucose deprivation can challenge cell viability by increasing the amount of cellular ROS, leading to cell death (150). PDAC copes with glucose deprivation by upregulating genes involved in ROS detoxification. Nuclear factor erythroid 2-related factor 2 (NRF2), a critical transcription factor regulating cellular antioxidant genes, is constitutively stabilized, enabling PDAC cells to cope with ROS (151). Additionally, PDAC cells can increase ROS detoxification by upregulating expression of isocitrate dehydrogenase 1 (IDH1), which supports the regeneration of NADPH required for cellular antioxidant capacity (150). Both processes are critical for progression of murine PDAC tumors, and clinical studies are underway to determine if limiting ROS detoxification can improve PDAC outcomes (NCT05209074).

That said, tolerance to nutrient stress does not necessarily require a robust cellular response. Another surprising strategy that PDAC may employ is to do nothing at all. Not responding to metabolic stress, or “phenotypic inertia,” allows PDAC cells to evade major antiproliferative and apoptotic responses to metabolic stress. Nutrient deprivation can trigger apoptotic cell death, but PDAC cells dampen apoptotic signaling under nutrient stress and simply fail to respond (13, 40, 152). Upregulation of antiapoptotic proteins is one mechanism by which PDAC cells overcome starvation-induced apoptosis (13). Induction of antiapoptotic proteins is critical for PDAC cells to survive in poorly perfused regions of tumors and drives the growth of murine PDAC tumors (13). Beyond apoptosis, “inertia” may play a general role in how tumors adapt to nutrient stress in the TME. Loss of epigenetic regulators in certain tumors induces “transcriptional numbness” in cancers, which prevents tumors from inducing stress-related genes that limit cancer cell growth (153). Indeed, mutations in epigenetic regulators are selected in nutrient-starved conditions in culture and in animal tumors (153). While the role of transcriptional numbness has not been examined in the context of PDAC, mutations in epigenetic regulators are frequent in advanced PDACs (154). Thus, future studies are warranted to determine if transcriptional numbness enables PDAC to tolerate nutrient stress in the TME.

Importantly, phenotypic inertia also contributes to other aspects of PDAC biology such as drug resistance. For instance, nutrient starvation-driven increases in antiapoptotic proteins not only allow PDAC to survive this stress, but also cause cross-resistance to chemotherapy agents like gemcitabine (13). Likewise, glucose deprivation, which forces cells to adapt by increasing their capacity for quenching ROS, significantly reduces PDAC cell sensitivity to gemcitabine — which is also associated with cytotoxic ROS production (150, 155, 156). Notably, dietary interventions that limit the ability of PDAC to tolerate cellular stress could serve as adjuvants to improve the efficacy of PDAC therapeutics. For example dietary interventions that increase glucose levels in PDAC improve the efficacy of chemotherapy regimens whose activity in part depends on generating cellular redox stress (156). In another example, ketogenic diets on their own have little effect in slowing PDAC progression in preclinical studies (98, 157). However, forced ketone oxidation impairs PDAC response to redox stress, and ketogenic diet has a notable effect in extending overall survival when combined with standard chemotherapy regimens that cause redox stress in PDAC tumors (157). Thus, understanding how PDAC becomes “numb” to the metabolic stress of the TME will not only help reveal how tumors adapt to nutrient stress, but can provide insight into pathological features of PDAC such as drug resistance.

## Conclusions and emerging areas of research

Limited and dysfunctional vasculature in PDAC has long led to the hypothesis that the PDAC TME is metabolically “hostile.” Recent advancements in tumor physiology and metabolomics have pinpointed which nutrients are limited in the TME, providing new insight into precisely what metabolic stresses occur in PDAC (22–24) (Figure 1). We now know that PDAC tumors are depleted of many nutrients, including oxygen, glucose, and several amino acids. The PDAC TME is also acidic and unexpectedly features many accumulated metabolites. However, analyses of nutrient levels in the PDAC TME are far from complete. Specific classes of nutrients that are critical for cancer metabolism, such as lipids, have not been systematically measured in the TME. Future work that more broadly characterizes nutrients in the PDAC TME will be critical to understand the metabolic challenges posed by the environment as well as the different substrates available to cancer cells to circumvent these obstacles.

Additionally, to fully understand how PDAC adapts to nutrient stress in the TME, much work remains to characterize nutrient levels across a diverse set of PDAC tumors and how these levels change over disease progression. First, while new methods for measuring nutrient levels in the TME have provided insight into metabolic stresses in PDAC, most of these measurements have been made (a) in animal models of PDAC, (b) from primary tumors, and (c) at one point of disease progression. Thus, future work examining TME nutrient levels across time and location will be required to have a complete picture of how TME metabolic stress evolves with PDAC disease progression. Importantly, efforts must also be made to measure TME nutrient levels in specimens of patients with PDAC, as most studies have been restricted to genetically homogenous animal models with unknown similarity to the metabolic stresses present in human disease. Additionally, little is known about whether there is substantial intratumoral heterogeneity in TME nutrient availability. Current TME nutrient measurements from interstitial fluid samples provide an average value of nutrient availability across the entire tumor. However, it is likely that many of these nutrients are not uniform in their distribution. Oxygen and pH concentrations in tumors are graded with distance from the nearest blood vessel (158). Determining if similar gradients exist for other nutrients will be key to understanding how TME metabolic stress affects PDAC biology.

Nevertheless, given these dramatic changes in nutrient availability within the TME, PDAC must adapt to grow and survive in this environment. The adaptations that PDAC requires to cope with TME nutrient stress can be classified into the categories proposed above. Already, some of these adaptations have been identified as potential therapeutic targets for restricting PDAC progression and are now being evaluated clinically (NCT05209074). Further work will be required to determine whether these nutrient features and metabolic dependencies are distinct to PDAC or are common features among similar solid malignancies. However, preliminary analyses suggest there are PDAC-specific challenges and adaptive mechanisms. For example, nutrient availability in the TME of PDAC and lung adenocarcinomas are distinct (35), suggesting that there are unique metabolic challenges in these two tumor types. Biochemical and genetic analysis suggests that many important PDAC metabolic dependencies are dispensable for lung tumor growth (16, 159). However, further work will be necessary to determine what other diseases experience similar metabolic stresses as PDAC and whether they activate similar adaptive mechanisms.

Finally, there is also limited knowledge about how multiple nutrient stresses in the PDAC TME interact and translate into functional dependencies for PDAC cells. Most studies of how nutrient stress affects PDAC cell biology involve modulation of a single metabolite in low-complexity cell culture models. While these studies have yielded fundamental insights into cell biology, ultimately, nutrient composition in the TME is much more complex. Many PDAC cell requirements identified from cell culture experiments with single nutrient stresses are dispensable for PDAC tumor progression due to the presence of additional protective factors in the TME. Advances in model systems and screening technology to uncover how multiple nutrient cues interact, such as new physiological media (160), and dispensing devices that can generate large libraries of cell culture conditions with varied levels of multiple nutrients (47, 161–163) will be key to determining how nutrient stresses interact within the TME. In addition, advancements in in vivo genetic screening will reveal critical dependencies of cancer cells normally hidden by standard culture experiments (16, 106, 107). Together, we expect these advances to reveal a more complete picture of both the metabolic challenges PDAC cells face in tumors as well as the adaptations they become reliant upon to survive. Additionally, this work hold promise to uncover new therapeutic opportunities for PDAC, which leverage the metabolic limitations intrinsic to the unique physiology of these tumors.

## Version 1 07/15/2025

Electronic publication

## References

[1] https://www.cancer.org/research/cancer-facts-statistics/all-cancer-facts-figures/2024-cancer-facts-figures.html.

[2] Kolbeinsson HM (2023). Pancreatic cancer: a review of current treatment and novel therapies. J Invest Surg.

[3] Jiang Y, Sohal DPS (2023). Pancreatic adenocarcinoma management. JCO Oncol Pract.

[4] Sherman MH, Beatty GL (2023). Tumor microenvironment in pancreatic cancer pathogenesis and therapeutic resistance. Annu Rev Pathol.

[5] Beatty GL (2021). The biological underpinnings of therapeutic resistance in pancreatic cancer. Genes Dev.

[6] Leppänen J (2019). Tenascin C, fibronectin, and tumor-stroma ratio in pancreatic ductal adenocarcinoma. Pancreas.

[7] Kahn BM (2021). The vascular landscape of human cancer. J Clin Invest.

[8] Hasselluhn MC (2024). Tumor explants elucidate a cascade of paracrine SHH, WNT, and VEGF signals driving pancreatic cancer angiosuppression. Cancer Discov.

[9] Olive KP (2009). Inhibition of Hedgehog signaling enhances delivery of chemotherapy in a mouse model of pancreatic cancer. Science.

[10] Sofuni A (2005). Differential diagnosis of pancreatic tumors using ultrasound contrast imaging. J Gastroenterol.

[11] Sakamoto H (2008). Utility of contrast-enhanced endoscopic ultrasonography for diagnosis of small pancreatic carcinomas. Ultrasound Med Biol.

[12] Jacobetz MA (2013). Hyaluronan impairs vascular function and drug delivery in a mouse model of pancreatic cancer. Gut.

[13] Sela Y (2022). Bcl-xL enforces a slow-cycling state necessary for survival in the nutrient-deprived microenvironment of pancreatic cancer. Cancer Res.

[14] Shukla SK (2017). MUC1 and HIF-1alpha signaling crosstalk induces anabolic glucose metabolism to impart gemcitabine resistance to pancreatic cancer. Cancer Cell.

[15] Yamamoto K (2020). Autophagy promotes immune evasion of pancreatic cancer by degrading MHC-I. Nature.

[16] Zhu XG (2021). Functional genomics in vivo reveal metabolic dependencies of pancreatic cancer cells. Cell Metab.

[18] Menjivar RE (2023). Arginase 1 is a key driver of immune suppression in pancreatic cancer. Elife.

[19] Metcalf KJ (2021). Leveraging microenvironmental synthetic lethalities to treat cancer. J Clin Invest.

[20] Chan N (2010). Contextual synthetic lethality of cancer cell kill based on the tumor microenvironment. Cancer Res.

[21] Kaelin WG (2005). The concept of synthetic lethality in the context of anticancer therapy. Nat Rev Cancer.

[22] Apiz Saab JJ, Muir A (2023). Tumor interstitial fluid analysis enables the study of microenvironment-cell interactions in cancers. Curr Opin Biotechnol.

[23] Cognet G, Muir A (2024). Identifying metabolic limitations in the tumor microenvironment. Sci Adv.

[24] Sullivan MR, Vander Heiden MG (2019). Determinants of nutrient limitation in cancer. Crit Rev Biochem Mol Biol.

[25] Jang C (2018). Metabolomics and isotope tracing. Cell.

[26] Sullivan MR (2019). Isolation and quantification of metabolite levels in murine tumor interstitial fluid by LC/MS. Bio Protoc.

[27] García-Cañaveras JC (2019). The tumor metabolic microenvironment: lessons from lactate. Cancer Res.

[28] Wagner M, Wiig H (2015). Tumor interstitial fluid formation, characterization, and clinical implications. Front Oncol.

[29] Lee SC-ES (2023). Longitudinal dynamics of the tumor hypoxia response: From enzyme activity to biological phenotype. Sci Adv.

[30] Li L (2023). Searching for molecular hypoxia sensors among oxygen-dependent enzymes. Elife.

[31] Koong AC (2000). Pancreatic tumors show high levels of hypoxia. Int J Radiat Oncol Biol Phys.

[32] Vaupel P (2021). Oxygenation status of malignant tumors vs. Normal tissues: Critical evaluation and updated data source based on direct measurements with pO2 microsensors. Appl Magn Reson.

[33] Hay N (2016). Reprogramming glucose metabolism in cancer: can it be exploited for cancer therapy?. Nat Rev Cancer.

[34] Kamphorst JJ (2015). Human pancreatic cancer tumors are nutrient poor and tumor cells actively scavenge extracellular protein. Cancer Res.

[35] Sullivan MR (2019). Quantification of microenvironmental metabolites in murine cancers reveals determinants of tumor nutrient availability. Elife.

[36] Gorovits N, Charron MJ (2003). What we know about facilitative glucose transporters: Lessons from cultured cells, animal models, and human studies. Biochem Mol Biol Educ.

[37] Jacobs SR (2008). Glucose uptake is limiting in T cell activation and requires CD28-mediated Akt-dependent and independent pathways. J Immunol.

[38] Nwosu ZC (2023). Uridine-derived ribose fuels glucose-restricted pancreatic cancer. Nature.

[39] Zhang J (2017). Cancer cell metabolism: the essential role of the nonessential amino acid, glutamine. EMBO J.

[40] Recouvreux MV (2020). Glutamine depletion regulates Slug to promote EMT and metastasis in pancreatic cancer. J Exp Med.

[42] Fung TS (2025). Arginine: at the crossroads of nitrogen metabolism. EMBO J.

[43] Apiz Saab JJ (2023). Pancreatic tumors exhibit myeloid-driven amino acid stress and upregulate arginine biosynthesis. Elife.

[44] Lee MS (2023). Ornithine aminotransferase supports polyamine synthesis in pancreatic cancer. Nature.

[45] Hardy TA, May JM (2002). Coordinate regulation of L-arginine uptake and nitric oxide synthase activity in cultured endothelial cells. Free Radic Biol Med.

[46] Closs EI (2004). Plasma membrane transporters for arginine. J Nutr.

[47] Chidley C (2024). A CRISPRi/a screening platform to study cellular nutrient transport in diverse microenvironments. Nat Cell Biol.

[48] Hsu DJ (2023). Arginine limitation drives a directed codon-dependent DNA sequence evolution response in colorectal cancer cells. Sci Adv.

[49] Yang C (2024). Arginine deprivation enriches lung cancer proteomes with cysteine by inducing arginine-to-cysteine substitutants. Mol Cell.

[50] Xue C (2023). Tryptophan metabolism in health and disease. Cell Metab.

[51] Newman AC (2021). Immune-regulated IDO1-dependent tryptophan metabolism is source of one-carbon units for pancreatic cancer and stellate cells. Mol Cell.

[52] Pataskar A (2022). Tryptophan depletion results in tryptophan-to-phenylalanine substitutants. Nature.

[53] Bonifácio VDB (2021). Cysteine metabolic circuitries: druggable targets in cancer. Br J Cancer.

[55] Parra M (2018). Vitamin B6 and its role in cell metabolism and physiology. Cells.

[56] He C (2024). Vitamin B6 competition in the tumor microenvironment hampers antitumor functions of NK cells. Cancer Discov.

[57] McIntyre CL (2023). Diet, nutrient supply, and tumor immune responses. Trends Cancer.

[58] Arner EN, Rathmell JC (2023). Metabolic programming and immune suppression in the tumor microenvironment. Cancer Cell.

[59] Michl J (2022). CRISPR-Cas9 screen identifies oxidative phosphorylation as essential for cancer cell survival at low extracellular pH. Cell Rep.

[60] Chen JL-Y (2008). The genomic analysis of lactic acidosis and acidosis response in human cancers. PLoS Genet.

[61] Corbet C (2016). Acidosis drives the reprogramming of fatty acid metabolism in cancer cells through changes in mitochondrial and histone acetylation. Cell Metab.

[62] Murthy D (2024). Cancer-associated fibroblast-derived acetate promotes pancreatic cancer development by altering polyamine metabolism via the ACSS2-SP1-SAT1 axis. Nat Cell Biol.

[63] Kimbrough CW (2015). Targeting acidity in pancreatic adenocarcinoma: multispectral optoacoustic tomography detects pH-low insertion peptide probes in vivo. Clin Cancer Res.

[64] Cruz-Monserrate Z (2014). Targeting pancreatic ductal adenocarcinoma acidic microenvironment. Sci Rep.

[65] Eng CH (2010). Ammonia derived from glutaminolysis is a diffusible regulator of autophagy. Sci Signal.

[66] Briggs KJ (2016). Paracrine induction of HIF by glutamate in breast cancer: EglN1 senses cysteine. Cell.

[67] Papandreou I (2006). HIF-1 mediates adaptation to hypoxia by actively downregulating mitochondrial oxygen consumption. Cell Metab.

[68] Kim JW (2006). HIF-1-mediated expression of pyruvate dehydrogenase kinase: a metabolic switch required for cellular adaptation to hypoxia. Cell Metab.

[69] Biffo S (2024). The crosstalk between metabolism and translation. Cell Metab.

[70] Mrnjavac N, Martin WF (2025). GTP before ATP: The energy currency at the origin of genes. Biochim Biophys Acta Bioenerg.

[71] Bartman CR (2023). Slow TCA flux and ATP production in primary solid tumours but not metastases. Nature.

[72] Jackson RJ (2010). The mechanism of eukaryotic translation initiation and principles of its regulation. Nat Rev Mol Cell Biol.

[73] Jewell JL (2013). Amino acid signalling upstream of mTOR. Nat Rev Mol Cell Biol.

[74] Ryoo HD (2024). The integrated stress response in metabolic adaptation. J Biol Chem.

[75] Wang EM (2019). Expression and clinical significance of protein kinase RNA-like endoplasmic reticulum kinase and phosphorylated eukaryotic initiation factor 2α in pancreatic ductal adenocarcinoma. Pancreas.

[76] Bielska AA (2022). Activating mTOR mutations are detrimental in nutrient-poor conditions. Cancer Res.

[77] Nofal M (2017). mTOR inhibition restores amino acid balance in cells dependent on catabolism of extracellular protein. Mol Cell.

[78] Palm W (2015). The utilization of extracellular proteins as nutrients is suppressed by mTORC1. Cell.

[79] Nofal M (2022). GCN2 adapts protein synthesis to scavenging-dependent growth. Cell Syst.

[80] Gerner EW, Meyskens FL (2004). Polyamines and cancer: old molecules, new understanding. Nat Rev Cancer.

[81] Yang S (2011). Pancreatic cancers require autophagy for tumor growth. Genes Dev.

[82] Yang A (2014). Autophagy is critical for pancreatic tumor growth and progression in tumors with p53 alterations. Cancer Discov.

[83] Perera RM (2015). Transcriptional control of autophagy-lysosome function drives pancreatic cancer metabolism. Nature.

[84] Campbell S (2021). Glutamine deprivation triggers NAGK-dependent hexosamine salvage. Elife.

[85] Kim PK (2021). Hyaluronic acid fuels pancreatic cancer cell growth. Elife.

[86] Tsai PY (2021). Adaptation of pancreatic cancer cells to nutrient deprivation is reversible and requires glutamine synthetase stabilization by mTORC1. Proc Natl Acad Sci U S A.

[87] Bott AJ (2019). Glutamine anabolism plays a critical role in pancreatic cancer by coupling carbon and nitrogen metabolism. Cell Rep.

[88] Blachier J (2023). L-asparaginase anti-tumor activity in pancreatic cancer is dependent on its glutaminase activity and resistance is mediated by glutamine synthetase. Exp Cell Res.

[89] Recouvreux MV (2024). Glutamine mimicry suppresses tumor progression through asparagine metabolism in pancreatic ductal adenocarcinoma. Nat Cancer.

[90] Halbrook CJ (2022). Differential integrated stress response and asparagine production drive symbiosis and therapy resistance of pancreatic adenocarcinoma cells. Nat Cancer.

[91] Pathria G (2019). Translational reprogramming marks adaptation to asparagine restriction in cancer. Nat Cell Biol.

[92] Lien EC, Vander Heiden MG (2019). A framework for examining how diet impacts tumour metabolism. Nat Rev Cancer.

[93] Kanarek N (2020). Dietary modifications for enhanced cancer therapy. Nature.

[94] Bose S (2020). The molecular link from diet to cancer cell metabolism. Mol Cell.

[95] Tajan M, Vousden KH (2020). Dietary approaches to cancer therapy. Cancer Cell.

[96] Maddocks ODK (2017). Modulating the therapeutic response of tumours to dietary serine and glycine starvation. Nature.

[97] Maddocks ODK (2013). Serine starvation induces stress and p53-dependent metabolic remodelling in cancer cells. Nature.

[98] Lien EC (2021). Low glycaemic diets alter lipid metabolism to influence tumour growth. Nature.

[99] Evan T (2022). The roles of intratumour heterogeneity in the biology and treatment of pancreatic ductal adenocarcinoma. Oncogene.

[100] Yuan S (2019). Cellular plasticity in cancer. Cancer Discov.

[101] Zhou X (2023). Persister cell phenotypes contribute to poor patient outcomes after neoadjuvant chemotherapy in PDAC. Nat Cancer.

[102] Hwang WL (2022). Single-nucleus and spatial transcriptome profiling of pancreatic cancer identifies multicellular dynamics associated with neoadjuvant treatment. Nat Genet.

[103] Shiau C (2024). Spatially resolved analysis of pancreatic cancer identifies therapy-associated remodeling of the tumor microenvironment. Nat Genet.

[104] Diaz-Ruiz R (2011). The Warburg and Crabtree effects: On the origin of cancer cell energy metabolism and of yeast glucose repression. Biochim Biophys Acta.

[105] Birsoy K (2014). Metabolic determinants of cancer cell sensitivity to glucose limitation and biguanides. Nature.

[106] Biancur DE (2021). Functional genomics identifies metabolic vulnerabilities in pancreatic cancer. Cell Metab.

[107] Uijttewaal ECH CRISPR-StAR enables high-resolution genetic screening in complex in vivo models. Nat Biotechnol.

[108] Sheehan C, Muir A (2022). The requirement for mitochondrial respiration in cancer varies with disease stage. PLoS Biol.

[109] Bennett N (2022). Primary and metastatic tumors exhibit systems-level differences in dependence on mitochondrial respiratory function. PLoS Biol.

[110] Martínez-Reyes I (2020). Mitochondrial ubiquinol oxidation is necessary for tumour growth. Nature.

[111] Weinberg F (2010). Mitochondrial metabolism and ROS generation are essential for Kras-mediated tumorigenicity. Proc Natl Acad Sci U S A.

[112] Kay RR (2021). Macropinocytosis: biology and mechanisms. Cells Dev.

[113] Olivares O (2017). Collagen-derived proline promotes pancreatic ductal adenocarcinoma cell survival under nutrient limited conditions. Nat Commun.

[114] Walsh AB, Bar-Sagi D (2001). Differential activation of the Rac pathway by Ha-Ras and K-Ras. J Biol Chem.

[115] Bar-Sagi D, Feramisco JR (1986). Induction of membrane ruffling and fluid-phase pinocytosis in quiescent fibroblasts by ras proteins. Science.

[116] Commisso C (2013). Macropinocytosis of protein is an amino acid supply route in Ras-transformed cells. Nature.

[117] Ramirez C (2019). Plasma membrane V-ATPase controls oncogenic RAS-induced macropinocytosis. Nature.

[118] Yao W (2019). Syndecan 1 is a critical mediator of macropinocytosis in pancreatic cancer. Nature.

[119] Lee SW (2019). EGFR-Pak signaling selectively regulates glutamine deprivation-induced macropinocytosis. Dev Cell.

[120] Garcia-Bermudez J (2022). Adaptive stimulation of macropinocytosis overcomes aspartate limitation in cancer cells under hypoxia. Nat Metab.

[121] Davidson SM (2017). Direct evidence for cancer-cell-autonomous extracellular protein catabolism in pancreatic tumors. Nat Med.

[122] Garcia-Bermudez J (2018). Aspartate is a limiting metabolite for cancer cell proliferation under hypoxia and in tumours. Nat Cell Biol.

[123] Kamphorst JJ (2013). Hypoxic and Ras-transformed cells support growth by scavenging unsaturated fatty acids from lysophospholipids. Proc Natl Acad Sci U S A.

[124] Sullivan LB (2018). Aspartate is an endogenous metabolic limitation for tumour growth. Nat Cell Biol.

[125] Sullivan LB (2015). Supporting aspartate biosynthesis is an essential function of respiration in proliferating cells. Cell.

[126] Birsoy K (2015). An essential role of the mitochondrial electron transport chain in cell proliferation is to enable aspartate synthesis. Cell.

[127] Kerk SA (2022). Metabolic requirement for GOT2 in pancreatic cancer depends on environmental context. Elife.

[128] Han X (2024). Cancer-associated fibroblasts maintain critical pancreatic cancer cell lipid homeostasis in the tumor microenvironment. Cell Rep.

[129] Auciello FR (2019). A stromal lysolipid-autotaxin signaling axis promotes pancreatic tumor progression. Cancer Discov.

[130] Sousa CM (2016). Pancreatic stellate cells support tumour metabolism through autophagic alanine secretion. Nature.

[131] Banh RS (2020). Neurons release serine to support mRNA translation in pancreatic cancer. Cell.

[132] Parker SJ (2020). Selective alanine transporter utilization creates a targetable metabolic niche in pancreatic cancer. Cancer Discov.

[133] Zhu Z (2020). Tumour-reprogrammed stromal BCAT1 fuels branched-chain ketoacid dependency in stromal-rich PDAC tumours. Nat Metab.

[134] Zhao H (2016). Tumor microenvironment derived exosomes pleiotropically modulate cancer cell metabolism. Elife.

[135] Francescone R (2021). Netrin G1 promotes pancreatic tumorigenesis through cancer associated fibroblast driven nutritional support and immunosuppression. Cancer Discov.

[136] Dalin S (2019). Deoxycytidine release from pancreatic stellate cells promotes gemcitabine resistance. Cancer Res.

[137] Halbrook CJ (2019). Macrophage-released pyrimidines inhibit gemcitabine therapy in pancreatic cancer. Cell Metab.

[138] Abt ER (2020). Metabolic modifier screen reveals secondary targets of protein kinase inhibitors within nucleotide metabolism. Cell Chem Biol.

[139] Yuan M (2022). Cancer-associated fibroblasts employ NUFIP1-dependent autophagy to secrete nucleosides and support pancreatic tumor growth. Nat Cancer.

[141] Goldman O (2023). Early infiltration of innate immune cells to the liver depletes HNF4α and promotes extrahepatic carcinogenesis. Cancer Discov.

[142] Tan CR (2014). Pancreatic cancer cachexia: a review of mechanisms and therapeutics. Front Physiol.

[143] Porporato PE (2016). Understanding cachexia as a cancer metabolism syndrome. Oncogenesis.

[144] Lee JH (2019). Branched-chain amino acids sustain pancreatic cancer growth by regulating lipid metabolism. Exp Mol Med.

[145] Mayers JR (2014). Elevation of circulating branched-chain amino acids is an early event in human pancreatic adenocarcinoma development. Nat Med.

[146] Neyroud D (2023). Blocking muscle wasting via deletion of the muscle-specific E3 ligase MuRF1 impedes pancreatic tumor growth. Commun Biol.

[147] Mason EF, Rathmell JC (2011). Cell metabolism: an essential link between cell growth and apoptosis. Biochim Biophys Acta.

[148] Hollinshead KER (2020). Respiratory supercomplexes promote mitochondrial efficiency and growth in severely hypoxic pancreatic cancer. Cell Rep.

[149] Izuishi K (2000). Remarkable tolerance of tumor cells to nutrient deprivation: possible new biochemical target for cancer therapy. Cancer Res.

[150] Vaziri-Gohar A (2022). Limited nutrient availability in the tumor microenvironment renders pancreatic tumors sensitive to allosteric IDH1 inhibitors. Nat Cancer.

[151] DeNicola GM (2011). Oncogene-induced Nrf2 transcription promotes ROS detoxification and tumorigenesis. Nature.

[152] Blanco FF (2016). The mRNA-binding protein HuR promotes hypoxia-induced chemoresistance through posttranscriptional regulation of the proto-oncogene PIM1 in pancreatic cancer cells. Oncogene.

[153] Loukas I (2023). Selective advantage of epigenetically disrupted cancer cells via phenotypic inertia. Cancer Cell.

[154] Lomberk G (2019). Emerging epigenomic landscapes of pancreatic cancer in the era of precision medicine. Nat Commun.

[155] Zarei M (2017). Posttranscriptional upregulation of IDH1 by HuR establishes a powerful survival phenotype in pancreatic cancer cells. Cancer Res.

[156] Vaziri-Gohar A (2023). Increased glucose availability sensitizes pancreatic cancer to chemotherapy. Nat Commun.

[157] Yang L (2022). Ketogenic diet and chemotherapy combine to disrupt pancreatic cancer metabolism and growth. Med.

[158] Mayers JR (2016). Tissue of origin dictates branched-chain amino acid metabolism in mutant Kras-driven cancers. Science.

[159] Helmlinger G (1997). Interstitial pH and pO2 gradients in solid tumors in vivo: high-resolution measurements reveal a lack of correlation. Nat Med.

[160] Zuber J, Palm W (2024). Modelling and deciphering tumour metabolism in CRISPR screens. Nat Rev Cancer.

[162] Sun X (2022). Modulating environmental signals to reveal mechanisms and vulnerabilities of cancer persisters. Sci Adv.

[163] Kochanowski K (2021). Systematic alteration of in vitro metabolic environments reveals empirical growth relationships in cancer cell phenotypes. Cell Rep.

